# MiR-338-3p regulates neuronal maturation and suppresses glioblastoma proliferation

**DOI:** 10.1371/journal.pone.0177661

**Published:** 2017-05-11

**Authors:** James R. Howe, Emily S. Li, Sarah E. Streeter, Gilbert J. Rahme, Edmond Chipumuro, Grace B. Russo, Julia F. Litzky, L. Benjamin Hills, Kyla R. Rodgers, Patrick D. Skelton, Bryan W. Luikart

**Affiliations:** 1 Department of Physiology and Neurobiology, Geisel School of Medicine at Dartmouth College, Lebanon, New Hampshire, United States of America; 2 Department of Genetics, Geisel School of Medicine at Dartmouth College, Lebanon, New Hampshire, United States of America; Lewis Katz School of Medicine at Temple University, UNITED STATES

## Abstract

Neurogenesis is a highly-regulated process occurring in the dentate gyrus that has been linked to learning, memory, and antidepressant efficacy. MicroRNAs (miRNAs) have been previously shown to play an important role in the regulation of neuronal development and neurogenesis in the dentate gyrus via modulation of gene expression. However, this mode of regulation is both incompletely described in the literature thus far and highly multifactorial. In this study, we designed sensors and detected relative levels of expression of 10 different miRNAs and found miR-338-3p was most highly expressed in the dentate gyrus. Comparison of miR-338-3p expression with neuronal markers of maturity indicates miR-338-3p is expressed most highly in the mature neuron. We also designed a viral “sponge” to knock down *in vivo* expression of miR-338-3p. When miR-338-3p is knocked down, neurons sprout multiple primary dendrites that branch off of the soma in a disorganized manner, cellular proliferation is upregulated, and neoplasms form spontaneously *in vivo*. Additionally, miR-338-3p overexpression in glioblastoma cell lines slows their proliferation *in vitro*. Further, low miR-338-3p expression is associated with increased mortality and disease progression in patients with glioblastoma. These data identify miR-338-3p as a clinically relevant tumor suppressor in glioblastoma.

## Introduction

In humans, neurogenesis has been observed in two regions of the adult brain: the subventricular zone of the lateral ventricles and the subgranular zone of the dentate gyrus in the hippocampus [1, 2]. Neurogenesis in the dentate gyrus has been linked to numerous phenomena, including but not limited to learning, memory, conditioning, and pattern separation [3]. Some animal models of depression demonstrate reduced dentate gyrus neurogenesis; neurogenesis is necessary for certain antidepressants to be effective [4, 5]. Developing a better understanding of the mechanisms underlying dentate gyrus neurogenesis could provide strategies to manipulate these neural processes for therapeutic gain.

Over the course of adult neurogenesis in the dentate gyrus, continuously dividing neuronal progenitor cells residing in the subgranular zone migrate as immature neurons to the granule cell layer of the dentate gyrus, where they differentiate into granule cells [6]. These neurons also functionally integrate into hippocampal circuitry by extending both axons and dendrites, subsequently forming synapses with mature neurons [7]. The transition of neurons from an immature to a mature state involves many changes to diverse cellular functions, one being cell cycle arrest. Long-term cell cycle arrest, the G_0_ or quiescent phase, is crucial for maintenance of the highly specialized mature neuronal phenotype. Failure of neurons to maintain the G_0_ phase is associated with numerous neurodegenerative diseases, such as Alzheimer’s and Parkinson’s disease [8–10].

Recent studies show microRNAs (miRNAs) play a key role in regulating neuronal gene expression, which can impact neurogenesis in the dentate gyrus [11]. These miRNAs include miR-132, which is most highly expressed in mature neurons, and miR-137, which inhibits synaptic integration and dendritic spine growth when knocked down [12, 13].

Neurogenesis is a highly activity-dependent phenomenon. Proliferation of newborn neurons in the dentate gyrus increases in response to direct hippocampal stimulation as well as processes stimulating the hippocampus, such as exercise, spatial learning, and an enriched environment [14–16]. If these newborn neurons do not receive sufficient stimulation, they become much less likely to survive to maturity [17]. Once these neurons begin forming synapses, successful integration into existing neurocircuits also depends on the level of stimulation they receive [18, 19].

Earlier studies identified 10 activity-dependent miRNAs significantly upregulated following pilocarpine-induced seizures [20]. To determine which of these miRNAs plays a role in neurogenesis, we developed a lentiviral sensor to detect miRNAs *in vivo*, and found significant miR-338-3p expression in the dentate gyrus of adult mice. Using immunohistochemistry, we determined miR-338-3p to be most highly expressed in mature neurons. *In vivo* miR-338-3p knockdown revealed that granule cells deficient in miR-338-3p sprout multiple primary dendrites and change their overall organization, increasing their number of dendrites and altering branching angles. We observed *in vivo* miR-338-3p knockdown created regions of cellular neoplasia resembling glioblastoma (GBM) in the dentate gyrus. Overexpressing miR-338-3p *in vitro* confirmed our findings with regards to neoplasia, significantly decreasing the proliferation rate of miR-338-3p-deficient GBM cell lines. Thus, we conclude miR-338-3p endogenously regulates maturation of neurons, and miR-338-3p loss-of-function could contribute to tumorigenesis.

## Results

### MiR-338-3p is expressed at high levels in the dentate gyrus

**Fig 1:**
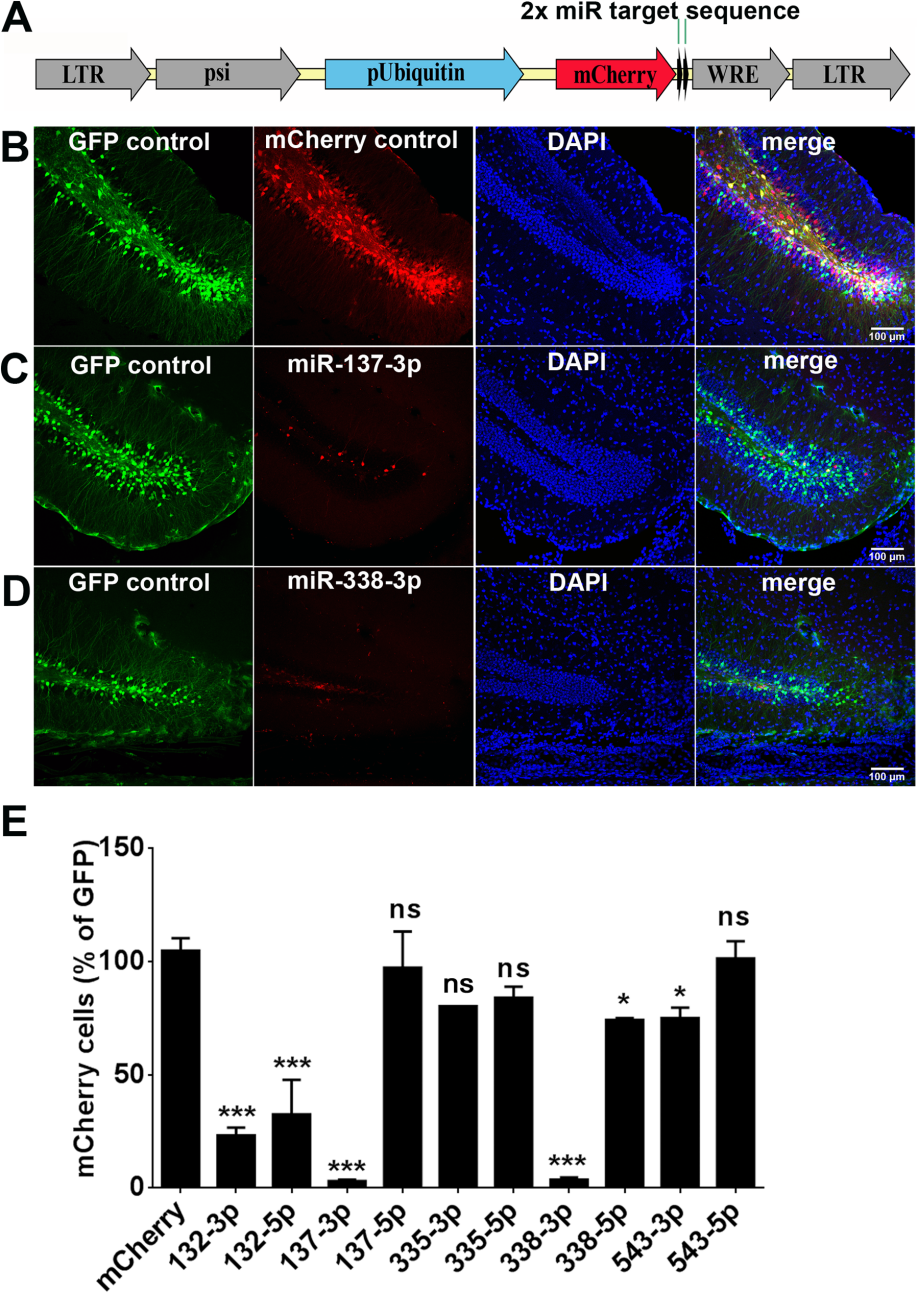
*In vivo* detection of selected miRNAs using an mCherry sensor. (A) Construction of the lentiviral vector, using an FUCW backbone and two target-complementary sequences immediately downstream of mCherry. (B) Co-injection of control GFP-expressing and mCherry-expressing viruses (equal titer) into the dentate gyrus of adult mice results in roughly equal infection rates; sections counter-stained with DAPI. (C) Co-injection of miR137-3p sensor (red) and control GFP-expressing virus. (D) Co-injection of miR338-3p sensor (red) and control GFP-expressing virus. (E) Expression levels of 10 different miRNAs in the dentate gyrus relative to control mCherry-expressing vector. *p<0.05, **p<0.01, ***p<0.001; one-way ANOVA, analyzed post-hoc using Tukey’s range test. Results show mean ± SEM.

We previously determined which miRNAs were most likely to affect neurogenesis by identifying miRNAs whose expression is induced by neuronal activity in a pilocarpine seizure model [20]. We selected the -3p and -5p species of the five most upregulated miRNAs for the current study. We designed a lentiviral sensor system to detect the miRNAs of interest via their binding to complementary mRNA sequences, which blocks translation. We achieved this by cloning two miRNA-complementary target sequences into the 3’ UTR of mCherry in a lentiviral vector (Fig 1A). Thus, endogenous miRNAs will bind the mCherry transcript’s target sequences, blocking its translation and reducing the level of mCherry fluorescence in cells expressing the miRNA of interest. Thus, if the cell expresses the miRNA of interest, mCherry fluorescence will be inhibited.

We co-injected equal titers of lentiviral controls containing green fluorescent protein (GFP) or mCherry without sensor into the dentate gyrus of adult mice (7–8 weeks). We use the GFP-expressing lentivirus to provide a baseline number of infected cells for comparison (Fig 1B). Next, we co-injected each of the 10 miRNA sensors and GFP control into the dentate gyri of adult mice (7–8 weeks). Qualitatively, we found that the miR-137-3p and miR-338-3p were the most strongly detected *in vivo* (Fig 1C and 1D). We subsequently quantified the expression level of each miRNA of interest by comparing the number of cells expressing the mCherry microRNA sensor to the number of cells expressing the GFP control (Fig 1E). We determined miR-137-3p and mir-338-3p were the most highly-expressed assayed miRNAs in the dentate gyrus, based on mean mCherry expression levels of 3.11 ± 0.53% (n = 3) and 3.68 ± 1.04% (n = 4) of cells, respectively, compared to GFP (Fig 1E, S1 Table). Based on these results, we decided to focus on miR-338-3p in subsequent experiments.

### MiR-338-3p is most highly expressed in mature neurons

To further characterize the temporal expression pattern of endogenous miRNAs in developing neurons, we immunostained murine dentate gyrus sections with neuronal maturation markers. We used Nestin, an intermediate filament protein, as a neural progenitor cell marker. We used doublecortin (DCX), a microtubule-associated protein expressed during neuronal migration, to mark neuronal precursor cells and newly differentiated neurons. Finally, we used Neuronal Nuclei Antigen (NeuN), a neuron-specific antigen localizing to the cell nucleus, to mark mature neurons. We compared the co-staining for these markers with a control virus constitutively expressing mCherry, the sensor for both the -3p and -5p transcripts of miR-338 or the sensor for the -3p and -5p transcripts of the previously characterized miR-132 [13].

**Fig 2:**
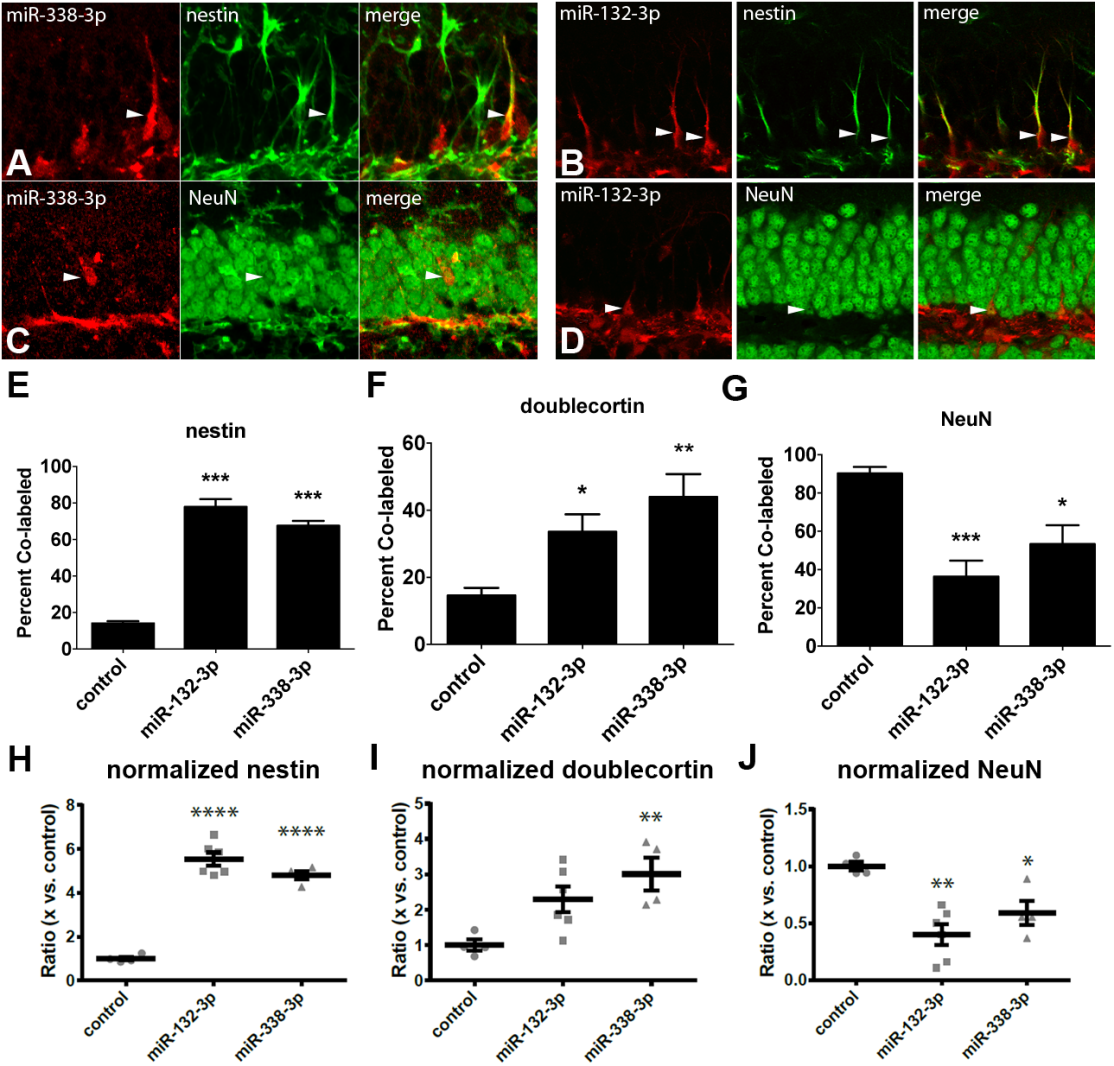
MiR-338-3p expression increases with maturity in dentate gyrus granule neurons. (A) Co-localization of nestin (arrowheads) with miR-338-3p sensor. (B) Co-localization of nestin (arrowheads) with miR-132-3p sensor. (C) Co-localization of NeuN (arrowheads) and miR-338-3p sensor. (D) Co-localization of NeuN (arrowheads) and miR-132-3p sensor. (E) Percentage of cells co-labeled with one of the sensors and nestin as compared to control mCherry virus. (F) Percentage of cells co-labeled with one of the sensors and doublecortin as compared to control mCherry virus. (G) Percentage of cells co-labeled with one of the sensors and NeuN as compared to control mCherry virus. (H) Ratio of cells co-labeled with either the miR-338-3p or miR-132-3p sensor and nestin as compared to control mCherry virus. (I) Ratio of cells co-labeled with either the miR-338-3p or miR-132-3p sensor and doublecortin as compared to control mCherry virus. (J) Ratio of cells co-labeled with either the miR-338-3p or miR-132-3p sensor and NeuN as compared to control mCherry virus. ns p>0.05, *p<0.05, **p<0.01, ***p<0.001, ****p<0.0001; one-way ANOVA, analyzed post-hoc using Tukey’s range test. Results show mean ± SEM.

In tissue injected with the mCherry control virus, 14.05 ± 1.19% (n = 4) of all mCherry-positive cells were nestin-positive, 14.60 ± 2.26% (n = 4) were DCX-positive, and 90.23 ± 3.37% (n = 4) were NeuN-positive (Fig 2E–2G, S2 Table). Some cells co-expressed DCX with nestin or NeuN, so the total proportions summed to greater than 100%. These results indicate the dentate gyrus is mostly comprised of mature neurons (NeuN), along with a smaller population of precursor cells (nestin) and newly differentiated neurons (DCX), which corresponds to prior findings examining the relative number of each cell population in the dentate gyrus, indicating the proportion of cells labeled by the sensor approximately reflects physiological proportions [21, 22].

We next quantified the percentage of nestin-, DCX-, and NeuN-positive cells labeled by the sensor in the dentate gyri of adult mice injected with either the miR-338-3p (Fig 2A and 2C) or the miR-132-3p sensor (Fig 2B and 2D). Of cells labeled by the miR-338-3p sensor, 67.50 ± 2.70% (n = 4) were nestin-positive, 43.97 ± 6.79% (n = 4) were DCX-positive, and 44.87 ± 5.87% (n = 4) were NeuN-positive (Fig 2E–2G). Of the miR-132-3p sensor-expressing cells, 77.84 ± 4.24% (n = 6) were nestin positive, 33.56 ± 5.22% (n = 6) were DCX-positive and 36.38 ± 8.33% (n = 6) were NeuN-positive (Fig 2E–2G). To illustrate the changes in expression for miR-338-3p and miR-132-3p specific to different points in neuronal maturity while compensating for any possible unforeseen biases toward specific cell types potentially induced by differential tropism, we described these percentages normalized to an mCherry control utilizing the same backbone (Fig 2H–2J). Compared to control, there was a 5.54 ± 1.29-fold (p<0.0001) and 4.80 ± 1.10-fold (p<0.0001) fold increase in the miR-132-3p and miR-338-3p sensor expression in nestin-positive cells (Fig 2H). Of all cells expressing the miR-132-3p sensor, there was a 2.30 ± 1.16-fold decrease (p<0.05) in co-labeling of DCX and the sensor compared to the control, and a 3.01 ± 1.61-fold decrease (p<0.01) in the miR-338-3p sensor-expressing neurons (Fig 2I). We found a 2.48 ± .22 (p<0.001) and 1.69 ± .27 (p<0.05) fold decrease in co-labeling of NeuN with miR-132-3p and miR-338-3p sensors, respectively (Fig 2J). These data indicate an increase in relative miR-338-3p expression with advancing neuronal maturity. Given the expression profile of miR-338-3p, we posit that it may have a role regulating the maturation of a proliferating neural precursor cell into a mature neuron.

### Granule cell morphology is altered by miR-338-3p knockdown

**Fig 3:**
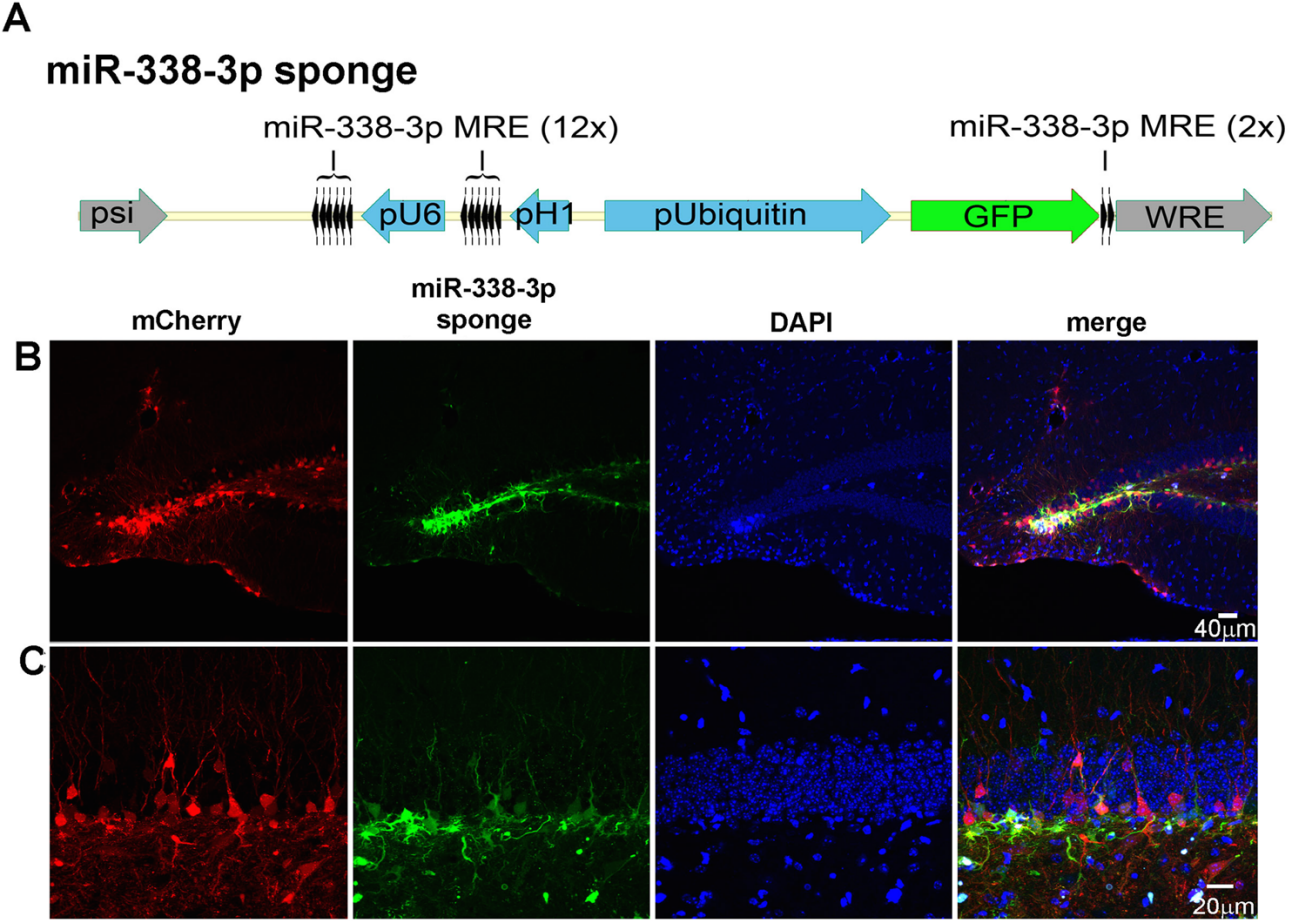
*In vivo* verification of miR-338-3p sponge efficacy. (A) Design of lentiviral miR-338-3p sponge with a sensor cassette. The miR-338-3p sensor cassette contains 2 perfectly complementary miR-338-3p target sequences downstream of GFP driven by the pUbiquitin promoter and the sponge cassette consists of 6 targets downstream of both the H1 and U6 promoters for a total of 2 sensor targets to sense miR-338-3p activity and 12 sponge targets to sequester endogenous miR-338-3p. (B) Low magnification images of dentate gyrus show the mCherry control and the miR-338-3p sponge exhibit similarly high levels of expression. (C) Images from (B), but under high magnification. This demonstrates the ability of the sponge cassette to sequester ligand away from the miR-338-3p targets expressed in the sensor cassette.

To evaluate the function of miR-338-3p *in vivo*, we generated a miR-338-3p lentiviral and retroviral ‘sponge’ to reduce activity of miR-338-3p by binding it and preventing the miRNA from interacting with its endogenous targets. The miR-338-3p sponge contains 6 targets downstream from both the U6 and H1 promoters, for a total of 12 sponge targets. We generated the sponge using the previously described sensor cassette that has 2 miR-338-3p targets downstream from GFP driven by a ubiquitin promoter, allowing simultaneous knockdown and detection of miR-338-3p expression *in vivo* (Fig 3A).

We determined effectiveness of the sponge *in vivo* by injecting a virus that expresses both the miR-338-3p sensor and miR-338-3p sponge cassettes. When infected with the miR-338-3p sensor, the average number of cells labeled per tissue section is 3.0 ± 2.19 cells/section (n = 6), while an equal titer of a control virus expressing only mCherry labeled 182.1 ± 12.81 cells/section, (n = 8) indicating strong regulation of the sensor by endogenous miR-338-3p (S3 Table). In contrast, when mice are injected with a virus expressing both the miR-338-3p sensor and the sponge, 101.5 ± 6.57 cells/section (n = 8) were labeled (S3 Table). This increase in the number of labeled cells infected with the miR-338-3p sponge shows the sponge effectively knocks down endogenous miR-338-3p. The efficacy of the miR-338-3p sponge was also demonstrated by co-injection with an equal titer of a control virus expressing mCherry alone (Fig 3B and 3C). We found that most cells labeled by the mCherry control were also labeled with the miR-338-3p sponge virus, but the control virus labeled a higher proportion of mature-appearing granule neurons compared to the sponge (Fig 3C). However, an analysis of the proportion of sponge-infected cells expressing nestin versus NeuN showed no significant changes to the overall relative maturity of sponge-infected cells (S1 Fig, S4 Table). These results indicate that the miR-338-3p sponge effectively knocked down miR-338-3p but does not alter the proportion that are nestin or NeuN positive.

**Fig 4:**
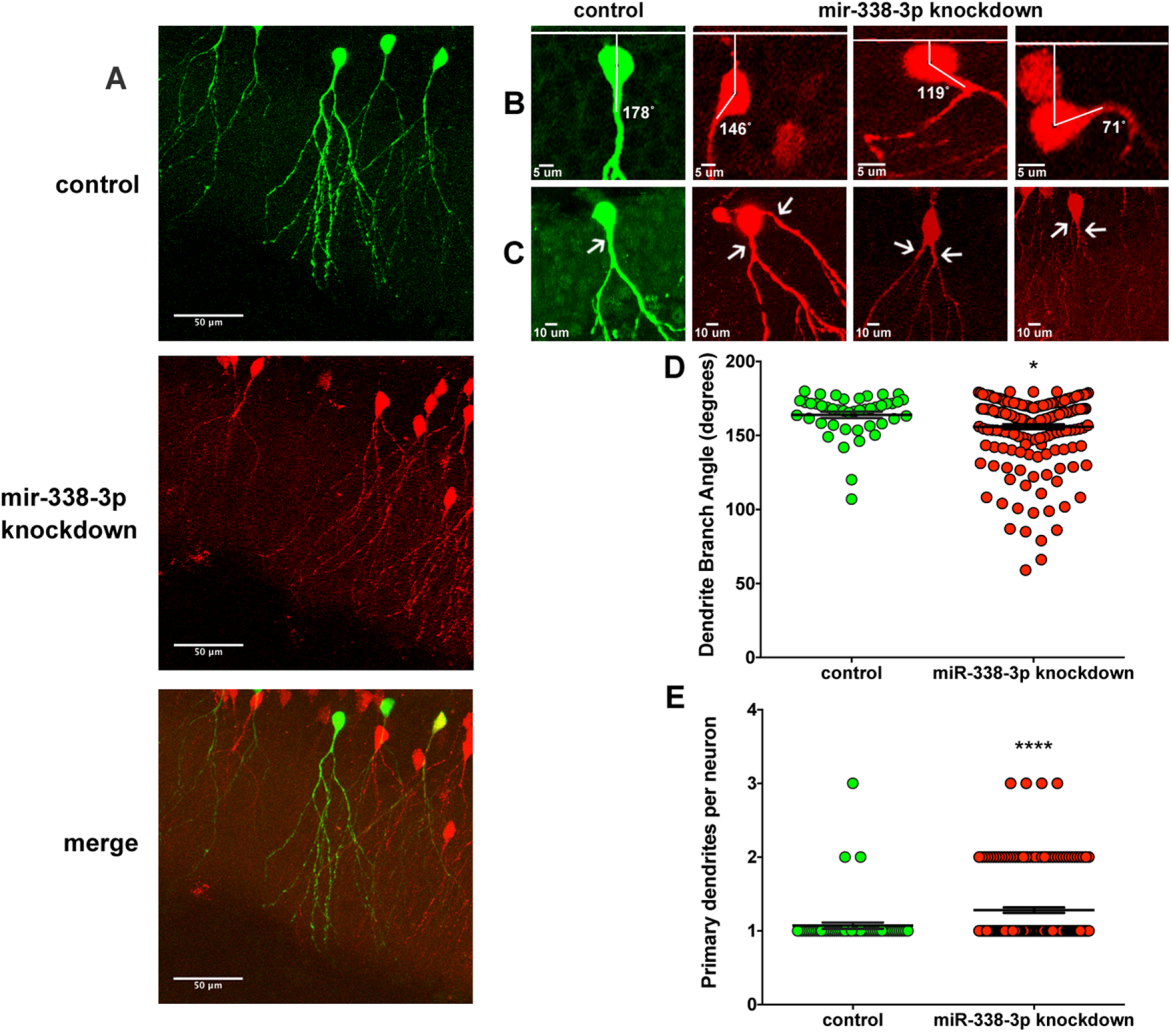
MiR-338-3p knockdown results in abnormal granule cell morphology in neonatal dentate gyrus. (A) Representative images of granule cells infected with the retroviral GFP control or the mCherry miR-338-3p sponge (red). (B) Granule neurons expressing the miR-338-3p sponge (red) displaying primary dendrites projecting at divergent angles from the soma compared to control neurons. (C) Granule cells infected with the control virus showing bipolar organization, while the miR-338-3p knockdown neurons (red) show multiple primary dendrites. (D) Branching angles of primary dendrites infected with the control vector or infected with the sponge. (E) Proportion of granule cells with multiple primary dendrites relative to all granule cells in both control and knockdown conditions. Each separate image was treated as an independent sample within the mouse of both the control and knockdown populations. *p<0.05, ****p<0.0001; t-test. Results show mean ± SEM.

To determine the function of miR-338-3p in the development of dentate gyrus granule neurons, we cloned a miR-338-3p sponge cassette into the pRubi retroviral backbone. Injection of this retrovirus results in specific infection of actively proliferating cells, allowing us to examine the development of newborn granule neurons *in vivo*. Two retroviruses, one expressing the sponge construct with mCherry and one expressing GFP only, were co-injected into the neonatal dentate gyrus. 21 days after injection, the granule layer was highly infected. The majority of labeled cells were either infected by the sponge or co-infected with both the sponge and the GFP control, and only a small minority was infected solely with the GFP control (Fig 4A). We limited our morphological analysis solely to granule neurons.

The granule neurons expressing the miR-338-3p sponge often displayed abnormal dendritic architecture with dendrites protruding from the soma at abnormal angles (Fig 4B). The granule cell is a bipolar neuron, projecting a single dendrite into the molecular layer oriented at a right angle to the hilus/granule cell border. To quantify the change in this angle caused by miR-338-3p knockdown, we created a line 90° from the granule layer/hilus border to the center of the soma and a second line along the axis of the dendrite, then measured the angle between the cell body and dendrite (Fig 4B). The granule cells expressing only the control vector had a mean branching angle of 163.9 ± 2.2° (n = 5), while the granule cells expressing the miR-338-3p sponge had a mean branching angle 155.8 ± 1.7° (n = 5), with a mean difference in branching angle of 8.0 ± 2.8°, indicating a significant deviation from the mean branching angle in miR-338-3p knockdown neurons (Fig 4D, S5 Table; p<0.01).

Some of the observed granule cells also appear to sprout multiple primary dendrites (Fig 4C). In neurons expressing the miR-338-3p sponge, there were 1.28 ± 0.04 (n = 5) primary dendrites/neuron on average, while control neurons averaged 1.07 ± .04 (n = 5) primary dendrites/neuron, showing a significant increase in the number of primary dendrites/neuron in miR-338-3p-deficient cells (Fig 4E, S6 Table; p<0.001). The majority of neurons with multiple primary dendrites had two, a minority had three, but none had four or greater. When neurons expressed the aberrant phenotype, the primary dendrites organized in one of two arrangements: one primary dendrite in the correct orientation and the other branching off at an angle approximately perpendicular to the other (Fig 4C, second image), or both branching out at angles close to the wild-type orientation, diverging away from the soma in opposite directions (Fig 4C, third and fourth image). Taken together, these morphological changes are indicative of aberrant granule neuron development when miR-338-3p expression is knocked down.

We did not observe any statistically significant changes in dendritic spine density, length, or head area (p>0.05, S7 Table). Analyses of soma size and length, number of dendritic nodes, number of dendritic termini, total dendrite length, and mean dendrite length all returned non-significant results (p>0.05, S8 Table). Sholl analyses of dendrite intersections and dendrite length yielded similarly non-significant results as well (p>0.05, S8 Table). Thus, we can infer that miR-338-3p knockdown only induces significant changes in the number of dendrites and their orientation, but not to the soma itself or to dendritic arborization and spine density.

### MiR-338-3p knockdown *in vivo* results in neoplasia

**Fig 5:**
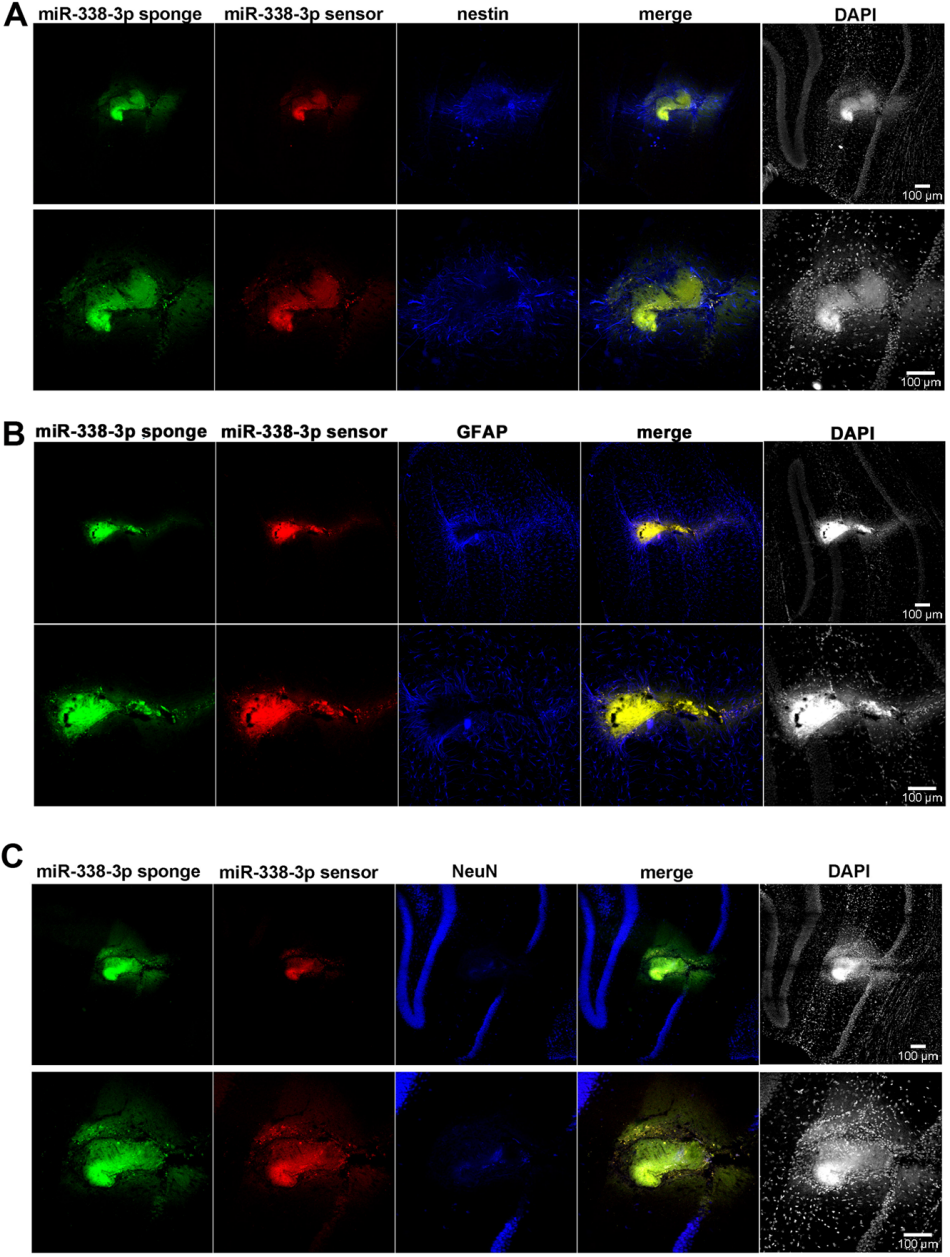
MiR-338-3p knockdown results in cellular neoplasia *in vivo*. Neoplasm infected with the miR-338-3p sensor (red) and sponge (green) and stained for (A) nestin (blue) as a marker for immature neurons, (B) GFAP (blue) as a marker for astrocytes, and (C) NeuN (blue) as a marker for mature neurons.

We noticed regions of cellular neoplasia after injection of either the retroviral or lentiviral miR-338-3p sponge. Co-injection of the lentiviral miR-338-3p sponge with the miR-338-3p sensor (n = 6) resulted in cellular neoplasia without exception at 7 days post-infection (DPI) in regions of high miR-338-3p sponge expression local to the injection region (Fig 5). When a lentiviral sponge construct for miR-132-3p with a similar overall design was injected instead (n = 4), no such regions of cellular neoplasia were observed at 7 DPI (S2 Fig).

To determine the composition of these abnormal cell clusters, we stained for nestin, glial fibrillary acidic protein (GFAP), and NeuN. Qualitatively, Nestin expression seemed to be almost entirely localized to the neoplasm’s margins, while mostly absent from the neoplasm’s center (Fig 5A). GFAP also appeared to be highly expressed at the margins of the neoplastic growths (Fig 5B). NeuN was observed at a much lower level than in the uninfected neurons surrounding the neoplasm. However, we did sporadically observe some NeuN-positive cells within regions of neoplasia (Fig 5C). Overall, these neoplasms appear to be generally composed of a ‘core’ expressing few biomarkers surrounded by a region of nestin- and GFAP-positive cells with small numbers of neurons interspersed throughout the two. This histological organization is reminiscent of GBM [23].

**Fig 6:**
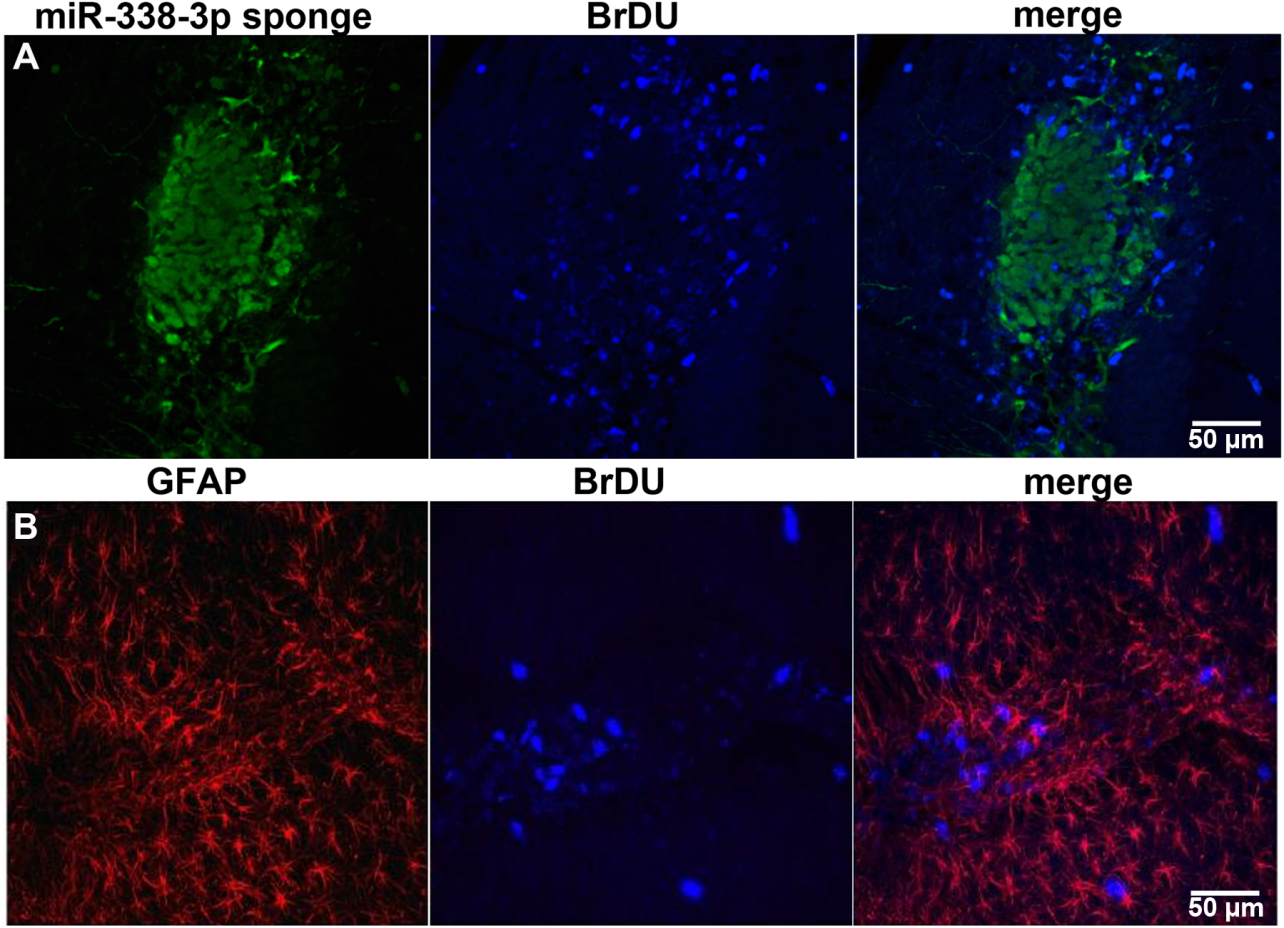
MiR-338-3p knockdown results in proliferation of GFAP-positive cells. (A) Expression pattern of of miR-338-3p sponge (green) and BrdU (blue), a marker of cellular proliferation, in a dentate gyrus neoplasm following miR-338-3p knockdown. (B) Co-localization of GFAP (red) and BrdU (blue) in dentate gyrus neoplasm following miR-338-3p knockdown.

To determine how these neoplasms proliferated, we injected 5-bromo-2’-deoxyuridine (BrdU) twice at 5 and 6 days after injection (n = 3) of the lentiviral miR-338-3p sponge and perfused the animals at 7 DPI (Fig 6). All mice injected displayed neoplasia local to the injection region. In observed neoplasms, few cells expressing the miR-338-3p sponge were BrdU-positive. We found most BrdU-positive cells on the edge of the neoplasm, with few in histologically normal regions, suggesting the cells on the margins of the neoplasm, adjacent to the sponge-infected cells on the margin, proliferate at an abnormally high rate (Fig 6A). These BrdU-positive cells tended to co-express GFAP as well, but not all BrdU-positive cells were also GFAP-positive, and these BrdU-expressing cells were a minority of GFAP-positive cells overall (Fig 6B). These data support the interpretation that there is a cell non-autonomous contribution of the miR-338-3p knockdown to cellular proliferation.

### MiR-338-3p overexpression inhibits proliferation in GBM cell lines

**Fig 7:**
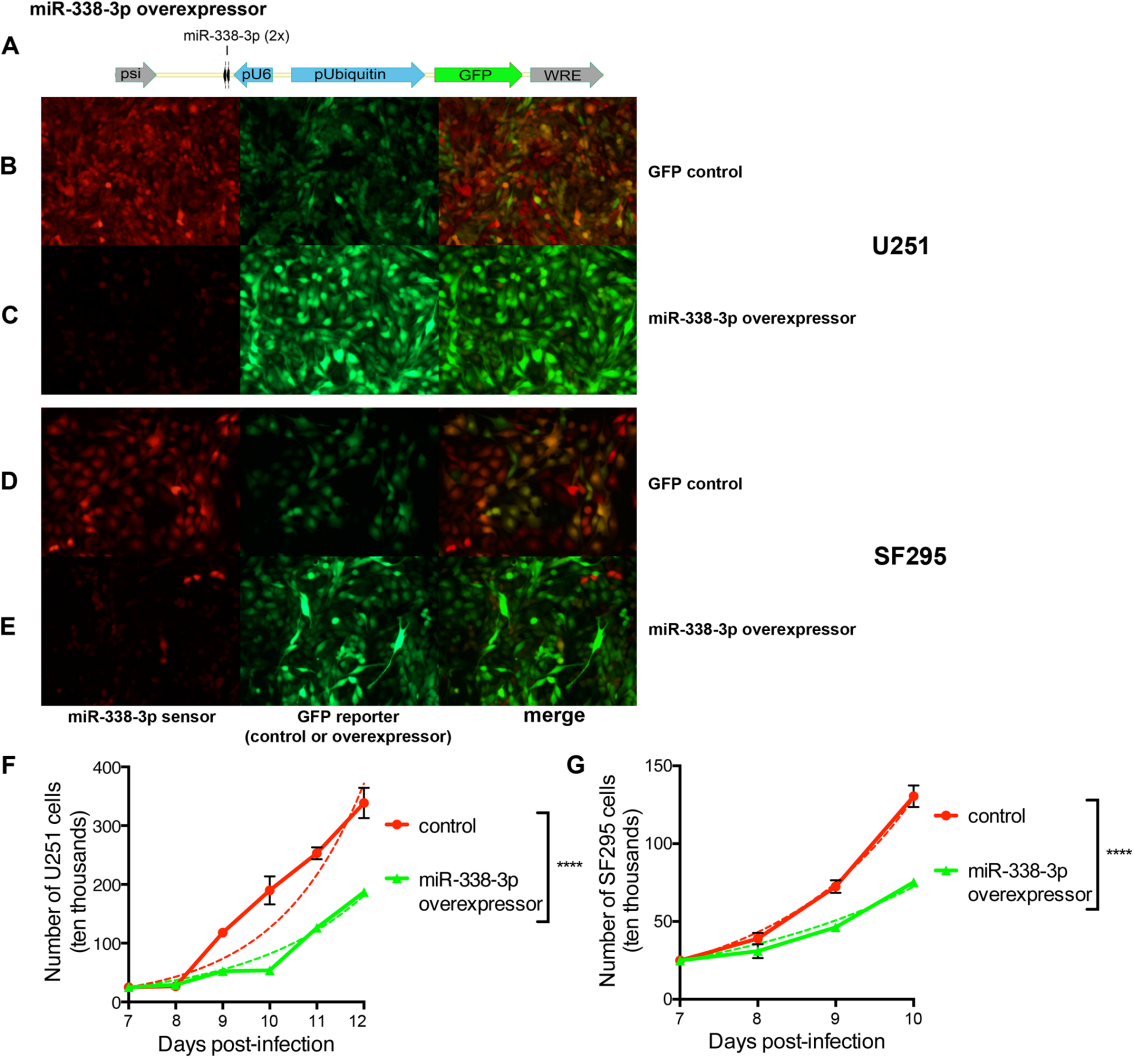
Overexpression of miR-338-3p decreases *in vitro* proliferation of GBM cells. (A) Construction of miR-338-3p overexpressor lentivirus containing a GFP-coding region to indicate expression along with two miR-338-3p transcripts downstream of the U6 promoter. (B) Endogenous expression of miR-338-3p in U251 GBM cells, as indicated by miR-338-3p sensor lentivirus (red) expression compared to control lentivirus expressing GFP-only. (C) Expression of miR-338-3p following infection with overexpressor virus in U251 GBM cells as indicated by miR-338-3p sensor (red). (D) Endogenous expression of miR-338-3p in SF295 GBM cells, as indicated by miR-338-3p sensor lentivirus (red) expression compared to control lentivirus expressing GFP-only. (E) Expression of miR-338-3p following infection with overexpressor virus in and SF295 GBM cells as indicated by miR-338-3p sensor (red). (F) Population growth kinetics of U251 GBM cells infected with an empty vector or miR-338-3p overexpressor (7–12 DPI). (G) Population growth kinetics of SF295 GBM cells infected with an empty vector or miR-338-3p overexpressor (7–10 DPI). Dotted lines in (F) and (G) fit theoretical population growth curves to the observed data, using the equation: Y = 25 × 2^t/DT^, where Y is the number of cells at time t, and DT is the doubling time. ****p<0.001; Pearson’s chi-squared test. Results show mean ± SEM.

To examine miR-338-3p function in GBM, we used an *in vitro* model of miR-338-3p activity. The U251 and SF295 GBM cell lines have previously been shown to be miR-338-3p deficient [24]. To determine whether miR-338-3p can directly impact cell division, we designed a lentiviral vector to overexpress miR-338-3p, expressing GFP under control of the ubiquitin promoter and two miR-338-3p sequences under control of the U6 promoter (Fig 7A). We verified both U251 and SF295 cells lack miR-338-3p by co-infecting them with miRNA-338-3p sensor virus and a control virus expressing GFP alone. The cells highly expressed mCherry compared to GFP, indicating low endogenous miR-338-3p expression (Fig 7B and 7D).

We ensured the overexpressor functioned as intended by co-infecting the two cell lines with the miR-338-3p sensor and either the miR-338-3p overexpressor or a control virus expressing only GFP (Fig 7B–7E). We noted a dramatic decrease in mCherry expression, indicating an increase in miR-338-3p expression in infected cells. From this, we concluded our overexpressor was an effective inducer of miR-338-3p expression in both cell lines.

To investigate the impact of miR-338-3p on cellular proliferation, we examined population growth kinetics for the U251 and SF295 cell lines infected with the miR-338-3p overexpressor. In both cell lines, miR-338-3p overexpression decreased the proliferation rate compared to control (Fig 7F and 7G, S9 Table). The control population was approximately double that of the miR-338-3p overexpressing population at the final time point in both cell lines. In U251, there were 34.1 ± 4.95 × 10^5^ cells in the control population and 18.7 ± 1.55 × 10^5^ cells in the miR-338-3p-overexpressing population at 12 DPI, with corresponding doubling times of 16.9 and 19.3 hours respectively, indicating an overall reduction in proliferation rate (Fig 7F; p<0.0001). In SF295, 13.05 ± 1.38 × 10^5^ control cells and 7.525 ± 0.275 × 10^5^ miR-338-3p overexpressing cells were present at 10 DPI, with respective doubling times of 12.6 and 14.7 hours (Fig 7G; p<0.0001). These results indicate miR-338-3p inhibits GBM proliferation *in vitro*.

### MiR-338-3p expression is associated with overall and disease-free survival in GBM patients

**Fig 8:**
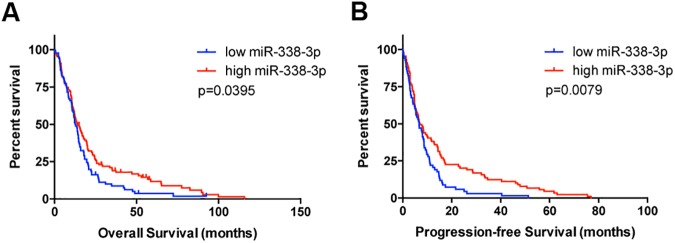
Outcomes in GBM patients based on miR-338-3p expression level. GBM patients with low mir-338-3p expression exhibit decreased overall and disease-free survival. Kaplan-Meier (A) survival and (B) disease-free survival curves of GBM patients grouped by mir-338-3p expression level. P-values determined by the log-rank test are indicated in the graphs.

We next determined whether miR-338-3p expression could be clinically significant. To do this, we examined data from The Cancer Genome Atlas (TGCA). Using data from 157 GBM patients with miRNA expression profiling of their tumors, we show that low expression of miR-338-3p corresponded to a decrease in overall and disease-free survival (Fig 8). These data suggest miR-338-3p is a clinically relevant miRNA with tumor suppressor properties.

## Discussion

These findings demonstrate a clear regulatory role for miR-338-3p in the development and proliferation of neurons in the dentate gyrus. Expression of miR-338-3p increases as a cell in the dentate gyrus matures, peaking in NeuN-positive mature neurons. This pattern of expression suggests a potential regulatory role in the development of a dividing neural precursor cell into a mature, terminally differentiated neuron. This potential activity of miR-338-3p is supported by its pattern of co-expression during maturation with miR-132, itself an inhibitory regulator of quiescence in other cell types [25].

The role of miR-338-3p in neuronal development is also apparent from our observations of aberrant morphology in miR-338-3p knockdown neurons. The two most obvious differences between wild type and miR-338-3p-deficient neurons are their increased likelihood to sprout multiple primary dendrites and subsequently branch off at deviant angles with respect to the soma. These changes indicate that miR-338-3p regulates proteins involved in neuronal polarization and neurite formation. Others have shown miR-338-3p regulates morphological polarity in other cell types by facilitating the activity of β-integrin [26]. It is possible that a similar mechanism could be responsible for aberrant dendrite placement on the soma. The process of neuronal polarization begins very early in the maturing neuron’s life, which is when miR-338-3p expression is increasing [27]. The mechanisms underlying initiation and termination of both neurite formation and neuronal polarization are not particularly well characterized, and verification of these hypotheses will require further study [28].

This study is the first to examine the effects of miR-338-3p on the morphology of dentate gyrus granule neurons, but other studies have described other changes to neural morphology induced by miR-338-3p, by a variety of molecular targets. MiR-338-3p regulates axonal outgrowth in cortical neurons through regulation of *Robo2*, an axonal guidance protein [29]. It also regulates axonal outgrowth in superior cervical ganglion neurons through *COXIV* and *ATP5G1*, two mitochondrial mRNAs active in oxidative phosphorylation [30]. MiR-338-3p is also expressed in spinal cord oligodendrocytes, where it exerts a related effect, inducing maturation and differentiation of precursors into mature oligodendrocytes by repressing *Sox5* and *Hes6*, two maturation-inhibiting transcription factors [31, 32]. Studying the effect of miR-338-3p on the expression of any of these genes or any in the same pathway could possibly provide a mechanistic understanding for how these primary dendrite changes are induced.

Surprisingly, both aberrant dendrite orientation and the presence of multiple primary dendrites have been previously described in studies of hippocampal neuron phenotypes in schizophrenia [33–36]. Additionally, miR-338 is downregulated in the brains of individuals suffering from schizophrenia [37]. Investigation of a potential causal link between miR-338-3p and schizophrenia could be promising.

Our morphological data from miR-338-3p knockdown indicate a relatively specialized role in regulating hippocampal neuron morphology. General loss of miRNA function through *Dicer1* ablation produces a wide range of changes in hippocampal dendrites, including but not limited to increased dendritic spine length, reduced dendritic arborization, and a bias toward arborization more distal to the soma [38, 39]. However, we did not observe any of these morphological changes in miR-338-3p knockdown neurons, indicating miR-338-3p is unlikely to contribute to miRNA regulation of these facets of hippocampal neuron morphology. The suite of changes we did observe were not studied in *Dicer1*-inactivated neurons, and examination of such changes in loss of general miRNA function could prove interesting as well.

In addition to changing the morphology of dentate granule neurons, miR-338-3p knockdown results in cellular neoplasia characterized by abnormal cellular proliferation. MiR-338-3p has previously been implicated in tumor suppression in hepatocarcinoma, gastric cancer, and brain cancer [40–44]. In this study, we found miR-338-3p knockdown leads to the formation of multiple neoplastic growths resembling GBM, showing a direct causal link between loss of miR-338-3p function and hippocampal tumorigenesis. Further, we find miR-338-3p expression in GBM cell lines decreases their proliferation *in vitro*. Another study shows reduced cell viability and increased apoptosis in certain glioma cell lines following overexpression of miR-338-3p, which may at least partially explain the change in proliferation we observe [40].

However, a recent study expressing miR-338-3p in U251 cells describes a different phenomenon, where induction of miR-338-3p expression has no effect on U251 proliferation [45]. These differences could be explained by divergent methodologies. Wang *et al*. examined changes during the first 96 hours of infection, while this study begins analysis 7 days after infection; differences in the time course of regulated genes’ activity could underlie differences observed between the two studies. Differences could also be explained by the manner of infection: while our vector contained only the miR-338-3p sequence, Wang *et al*. uses a longer sequence surrounding miR-338-3p. Regulatory elements within the sequence or miRNA processing differences could also contribute to the difference in effects observed.

We also determined miR-338-3p expression does have an impact on GBM severity in patients. Our analysis of the TCGA database indicates that when GBM lack miR-338-3p, they become more aggressive and more lethal. While miR-338-3p is instrumental to tumor suppression, we do not know its exact mechanism. In this study, we have determined that miR-338-3p inhibits cellular proliferation in GBM cell lines, mirroring the results of a similar analysis done on the role of miR-338-3p in colorectal carcinoma implicating loss of *SMO* inhibition [44]. It is possible these two tumor types could be suppressed via the same method of regulation. Future studies are required to understand whether miR-338-3p operates through a similar mechanism in GBM.

Upon histological examination of the tumor, it is clear that not every neoplastic cell is miR-338-3p deficient, but rather the core lacks miR-338-3p while cells at the periphery of the neoplasm maintain relatively normal levels of miR-338-3p expression. Interestingly, a similar pattern of miR-338-3p expression was found in the tumors of 15 GBM patients [46]. Further, the proliferating BrdU-positive cells in the neoplasms we describe appear to be those peripheral cells not expressing low levels of miR-338-3p. Such a pattern of proliferation suggests a potential cell-nonautonomous regulatory mechanism, which has not described previously. This result is unexpected, based on our other data, and warrants further study. One prior study in pituitary adenoma describes a mechanism of tumor suppression via regulation of *Pttg*, a paracrine growth factor, in pituitary adenoma. However, this study did not examine *in vivo* proliferation, and the gene’s expression has not been described in the hippocampus thus far [47].

Regulation of *CyclinD1* expression has been similarly implicated as a potential mechanism by which miR-338-3p affects cell proliferation [48]. CyclinD1 and associated CDKs may act as a “switch” in the cell cycle, and their activity is inhibited in terminally differentiated neurons but high in the embryonic nervous system [48–51]. Furthermore, CyclinD1 and these CDKs may control angles of neurite formation in hippocampal granule cells [51]. Thus, the phenotypes observed in this study are consistent with the finding that miR-338-3p regulates CyclinD1. Another possibility is that these changes are induced by loss of inhibition of *PREX2a*, another target of miR-338-3p [43]. A phenotype with multiple primary dendrites is associated with loss of *Pten*, which inhibits the PI3K/AKT pathway [52]. PREX2a inhibits Pten activity itself, and the phenotype resulting from loss of *PREX2a* regulation by miR-338-3p should resemble the one resulting from loss of *Pten*. Prior studies have already shown miR-338-3p’s inhibition of *PREX2a* is crucial in suppression of neuroblastoma proliferation [43].

In this study, we identified a regulatory role for miR-338-3p in neuronal proliferation, maturation, and neurite outgrowth and organization in the dentate gyrus, while also acting as a tumor suppressor *in vivo*. While we did observe physiological changes in the neuron, the mechanisms by which miR-338-3p activity regulates these processes in the dentate gyrus are not yet fully characterized. An important topic for further research will be the determination of which pathways are responsible for the myriad changes observed in the miR-338-3p-deficient hippocampal neuron. However, our results in GBM are encouraging, and could indicate GBM therapeutics targeting miR-338-3p could be a promising line of inquiry.

## Materials and methods

### Subjects

All procedures were approved by the Institutional Animal Care and Use Committee at the Geisel School of Medicine at Dartmouth College and conformed to federal, state, local, and Association for Assessment and Accreditation of Laboratory Animal Care standards, under Protocol Numbers 11-01-01 and 00002030(a). All mice used were of the C57BL/6J genetic background, obtained from The Jackson Laboratory. Morphology experiments used neonates (postnatal day 7) and all others used adults (7–8 weeks of age). Experiments used mixed-sex groups of mice with equivalent numbers of each sex. Animals were housed in a vivarium on a 12 hour light/dark cycle with food and water provided *ad libitum*.

### Reporter and overexpressor virus design and creation

To generate the miRNA reporter viruses, two perfect miRNA targets were cloned into the EcoR1 site in the 3’UTR of mCherry in the FUCW viral vector [13]. Complementary oligonucleotides containing two perfect target sequences for that miRNA flanked by overhangs for EcoR1 cloning sites, were designed and ordered from Integrated DNA Technologies. Target sequences were 100% homologous to the reverse complement of the mature miR sequence as listed on miRBase (www.mirbase.org). Each pair of complimentary oligonucleotides were annealed and ligated into the digested FUCW, using NEB Quick Ligase. To generate the miR-338-3p overexpressor virus, the mature miR-338-3p sequence with a canonical stem loop (5’- TTCAAGAGA-3’) was cloned into the BbsI/BglII site of pCMVU6, and the U6 promoter and miR338-3p sequence was excised and placed into the PacI/BstB1 site of the lentiviral FUGW vector. Colonies were sequenced to verify that the insert containing target sequences was successfully ligated into FUCW in the desired direction. The miR-338-3p sponge was constructed by inserting 6 perfect miR-338-3p target sequences downstream of the U6 promotor and 6 perfect miR-338-3p target sequences downstream of the H1 promotor via complimentary overhangs to Bbs1/BglII. This U6 cassette was placed into the FUGW lentiviral vector via PacI/BstBI and the H1 cassette was placed into the PacI site. The sponge cassette was transferred into the retroviral pRubi backbone via ligation into the BstBI and BamHI sites of redRubi. All DNA was collected using the NucleoBond Xtra Maxi protocol (Macherey-Negel).

### Viral packaging

FUCW-miR vectors, along with pCMVΔ8.9 and pVSV-g, were transfected into HEK 293-FT cells. HEK 293-FT cells were sustained with media composed of Iscove’s modified Dulbecco’s medium (IMDM) (high glucose) (Gibco 12440–053), 10% fetal bovine serum (FBS), 0.1 mM MEM Non-Essential Amino Acids, 2mM L-glutamine, 1% Pen-Strep and 500 μg/ml Geneticin® (only used during growth). One day prior to transfection, 293-FT cells were split to a concentration of 2.5–3.0 x 10^6^ cells/10 centimeter plate. To one polystyrene tube, 40 μl transfer vector, 26 μl pCMVΔ8.9, 18 μl pVSV-g, 1720 μl H_2_O and 200 μl 2.5M CaCl_2_ were added and gently mixed. The contents of this tube were slowly added to a second polystyrene tube containing 2000 μl 2xHBS (281 mM NaCl, 50 mM HEPES, 1.5 mM Na_2_HPO_4_ heptahydrate monobasic, pH to exactly 7.0). After leaving this tube in the dark for 30 minutes, 1 mL of the mixture was added to each of four separate plates of HEK 293-FT cells. After 24 hours, the plates were refreshed with new media, containing 2% FBS. At 48 and 72 hours after transfection, viral particles were collected, spun at 2000g for 10 minutes, and then filtered through a 0.45 μm PES low protein binding syringe filter. To concentrate the viral particles, a 5x PEG6000 (8% PEG6000, 0.3M NaCl) solution was added to the filtrate, and the solution was incubated at 4°C for 12 hours before being spun at 2500g for 45 minutes. The pellet was then resuspended in PBS and the virus stored at -80°C until injection.

### Stereotactic injections

Mice were anesthetized using an isoflurane gas system (Veterinary Anesthesia Systems Co.) with 4% isofluorane. The mice were secured in a Stoelting lab stereotaxic frame and continued to receive 2% isoflurane through a gas nose cone. In order to deliver the virus into the dentate gyrus of adults, a one-inch incision was made in the scalp, and holes were drilled through the skull (± 1.1 mm lateral, -1.9 mm anteroposterior, -2.5–2.3 mm ventral from bregma). In neonates, incisions were made directly into the skull (± 1.3 mm lateral, +1.55 horizontal, -2.3–2.0 mm ventral from lambda) Using a Stoelting Quintessential Sterotaxic Injector and a 10μl Hamilton syringe, up to 2 μl of lentivirus was injected into each hemisphere. The syringe was left in place for 2 minutes after injection before being slowly withdrawn. The scalp incision above the injection site was sutured; mice received post-operative topical lidocaine, betadine, and antibiotic ointment at the incision site as well as a peritoneal injection of ketaprofen in saline, and then were placed in a recovery chamber until they regained consciousness. Mice were examined and weighed each day up to a week post-operation, and care (including but not limited to re-suturing, topical anesthetic, and antibiotics) was provided *pro re nata*.

### Histology and BrdU labeling

To determine the effect of miR-338-3p on cellular proliferation, some mice were given an intraperitoneal injection of BrdU (150mg/kg) in 0.9% sterile saline solution 5 and 6 days post-viral injection (DPI). Mice were then perfused 7 DPI, 24 hours after the second BrdU injection. Mice used for morphological analysis were perfused 21 DPI. Mice were deeply anesthetized with 2% avertin, and were intracardially perfused with cold PBS+4% sucrose for approximately 5 minutes, followed by a solution of 4% PFA (paraformaldehyde) in PBS+4% sucrose for approximately 15 minutes. Each brain was post-fixed in 4% PFA in PBS+4% sucrose solution overnight. For all procedures with the exception of morphological analysis, 50 μm thick coronal sections were cut using a Leica 1200S vibratome. Free-floating sections were permeabilized for 30 minutes with PBS-T, followed by 2 quick rinses with PBS-T. For BrdU staining, sections were treated with 1.5M HCl in PBS for 30 minutes, then heat treated with sodium citrate for 30 minutes, cooled at room temperature for 20 minutes, followed by 1 hour of blocking with 10% DHS/PBS-T. Sections were treated with primary antibodies in PBS with 2% donkey serum and 0.3% Triton X-100 overnight at 4 degrees with a combination of no more than three of the following primary antibodies overnight: rat anti-BrdU (1:200 Serotec/Bio-Rad), Chicken anti-GFP (1:2000 Abcam), mouse anti-Nestin (1:100 Chemicon MAB353), rabbit anti-GFAP (1:5000 Advanced Immunochemicals), mouse anti-NeuN (1:500 Abcam), and rabbit anti-mCherry (1:2000, Abcam). The following day, sections were washed 3 times every 15 minutes, followed by 2 quick rinses with PBS-T. Primary antibodies were detected using the following secondary antibodies in a 1:200 dilution: Dylight 647 anti-rat, Alexa488 anti-chicken, Cy3 anti-mouse, and Cy3 anti-rabbit (all Jackson Immunoresearch). Sections were incubated overnight before mounting.

### Measurement of granule cell morphology

All neurons were measured using ImageJ. To determine the angle of the neurons’ primary dendrites, the border between the granule layer and the molecular layer was traced. All granule cells with clearly defined somas and primary dendrite branch points were analyzed. The centers of all analyzed neurons’ somas were determined and marked with a black point, and a line orthogonal to the border between the two layers was made from the border to the center of the soma. A line was then made from the center of the soma to the point where the primary dendrite branched off of the soma. The angle created by the two lines, with the soma’s center as the vertex, was then measured. To determine the proportion of neurons with multiple primary dendrites, multiple images were captured from sections of each dendrite, and the number of neurons with multiple primary dendrites was divided by the total number of neurons in the image for each condition. Total dendritic arborization and soma size were also analyzed using NeuroLucida (MBF Bioscience).

### Preparation of *in vitro* samples and analysis of cellular proliferation

The U251 and SF295 cell lines were generously donated by the Israel laboratory at the Geisel School of Medicine at Dartmouth. Cells were grown in triplicate in 10 cm plates (Corning Incorporated) in Dulbecco’s Modified Eagle Medium (Life Technologies) containing 10% fetal bovine serum and 1% penicillin/streptomycin. Cells were counted using a hemocytometer, and once each plate reached 200,000 cells, the cells were infected with either the miR-338-3p overexpressor virus or a control virus expressing only the fluorophore at a multiplicity of infection of 20. The cells were then left to grow for 7 days to fully express the viral product. At 7 days post-infection, the cells were re-plated at 25,000/plate and counted every 24 hours for the next 5 days, and visualized using an Olympus IX-73 fluorescence microscope with a 10x objective lens. We were able to perform a cell count for the U251 line at all time points, but SF295 cells became overconfluent at 96 hours. Despite this, growth curves and proliferation rates could be calculated from the data at significance for both cell lines.

### Imaging and image analysis

Histological sections stained with antibodies for NeuN, Nestin, doublecortin, or BrdU were imaged using a Zeiss LSM 510 confocal microscope (40X oil lens) and quantified over the maximum possible analyzable depth. All other sections were imaged with the 20X lens and analyzed over a depth of 5–16μm and an area of 650.5 μm by 650.5 μm. A 63x oil lens with 3x digital zoom was used to take images of dendrite segments of neurons immunostained with Alexa488. Point-spread functions (PSFs) were generated for the image using the PSF Generator plugin for ImageJ, and then images were deconvolved using a Richardson-Lucy function based in the DeconvolutionLab plugin for ImageJ. The NeuronStudio program was then used to automatically quantify deconvolved segments of dendrites for spine density, spine length, and spine head diameter. Between one to three images were quantified per injected mouse. To correct for variability in overall staining intensity, the background fluorescence intensity of the entire image was subtracted from the intensity of the individual cell of interest. Using ImageJ, all cells labeled with FUCW or both FUCW and antibody were counted. Between one to three images were quantified per injected mouse.

### TGCA data

TCGA data was mined as previously described using Level 3 data from Chin L, *et al*. [53, 54]. A Z-value of -0.4 or less defined the mir-338-3p low subgroup. The remaining patients constituted the mir-338-3p high subgroup.

### Statistical analysis

For the *in vitro* proliferation assays, we performed Poisson regression in Stata. Significance was determined using Pearson’s chi-squared test, where α = 0.05. Figures were created using GraphPad Prism. For histological analysis, t-tests or one-way ANOVA analyzed post-hoc via Tukey’s range test were used unless otherwise indicated. We accounted for individual differences in counts and measurements of neurons between mice using previously described models [41]. Significance was set at p<0.05 for all analyses. For TGCA data analysis, GraphPad Prism 6 was used for statistical analysis. Log-rank (Mantel-Cox) was used to perform significance analysis on survival curves with 2 groups.

## Supporting information

S1 Fig MiR-338-3p knockdown does not alter relative maturity of affected cells. A. Representative images displaying co-localization of nestin (blue) with an mCherry control virus (red) and the miR-338-3p sponge virus (green). B. Representative images displaying co-localization of nestin (blue) with an mCherry control virus (red) and the miR-338-3p sponge virus (green). C. Ratio of co-labeling of cells by nestin and either the miR-338-3p sensor (n = 4) or the miR-338-3p sponge (n = 4) normalized to respective co-expressed mCherry and nestin co-labeling. D. Ratio of co-labeling of cells by NeuN and either the miR-338-3p sensor (n = 4) or the miR-338-3p sponge (n = 5) normalized to respective co-expressed mCherry and NeuN co-labeling. ns p>0.05, one way ANOVA. Results show mean ± SEM.
(TIF)

S2 Fig MiR-132-3p knockdown does not result in glioblastoma. A. Design of lentiviral miR-132-3p sponge with a sensor cassette, using the same vector backbone as the miR-338-3p sponge. The miR-132-3p sensor cassette contains 2 perfectly complementary miR-132-3p target sequences downstream of GFP driven by the pUbiquitin promoter and the sponge cassette consists of 6 targets downstream of both the H1 and U6 promoters for a total of 2 sensor targets to sense miR-132-3p activity and 12 sponge targets to sequester endogenous miR-132-3p. B. Dentate gyrus histology at 7 DPI after miR-132-3p sponge injection. The miR-132-3p sponge knocks down miR-132-3p expression in a subset of dentate gyrus neurons, allowing expression of the GFP sensor construct (green). No neoplastic glioblastoma-like histology was observed.
(TIF)

S1 Table Baseline sensor expression. (CSV)

S2 Table Sensor co-expression with maturity biomarkers. (CSV)

S3 Table MiR-338-3p sponge validation. (CSV)

S4 Table Sponge co-expression with maturity biomarkers. (CSV)

S5 Table Dendritic branch angles. (CSV)

S6 Table Primary dendrite quantities. (CSV)

S7 Table Dendritic spine properties. (CSV)

S8 Table Dendritic arborization. (CSV)

S9 Table GBM proliferation. (CSV)
