# Supplementary figures and images for: MiR-338-3p regulates neuronal maturation and suppresses glioblastoma proliferation
Source: PLoS One. 2017 May 11;12(5):e0177661. doi: 10.1371/journal.pone.0177661 (PMC5426787; doi:10.1371/journal.pone.0177661)

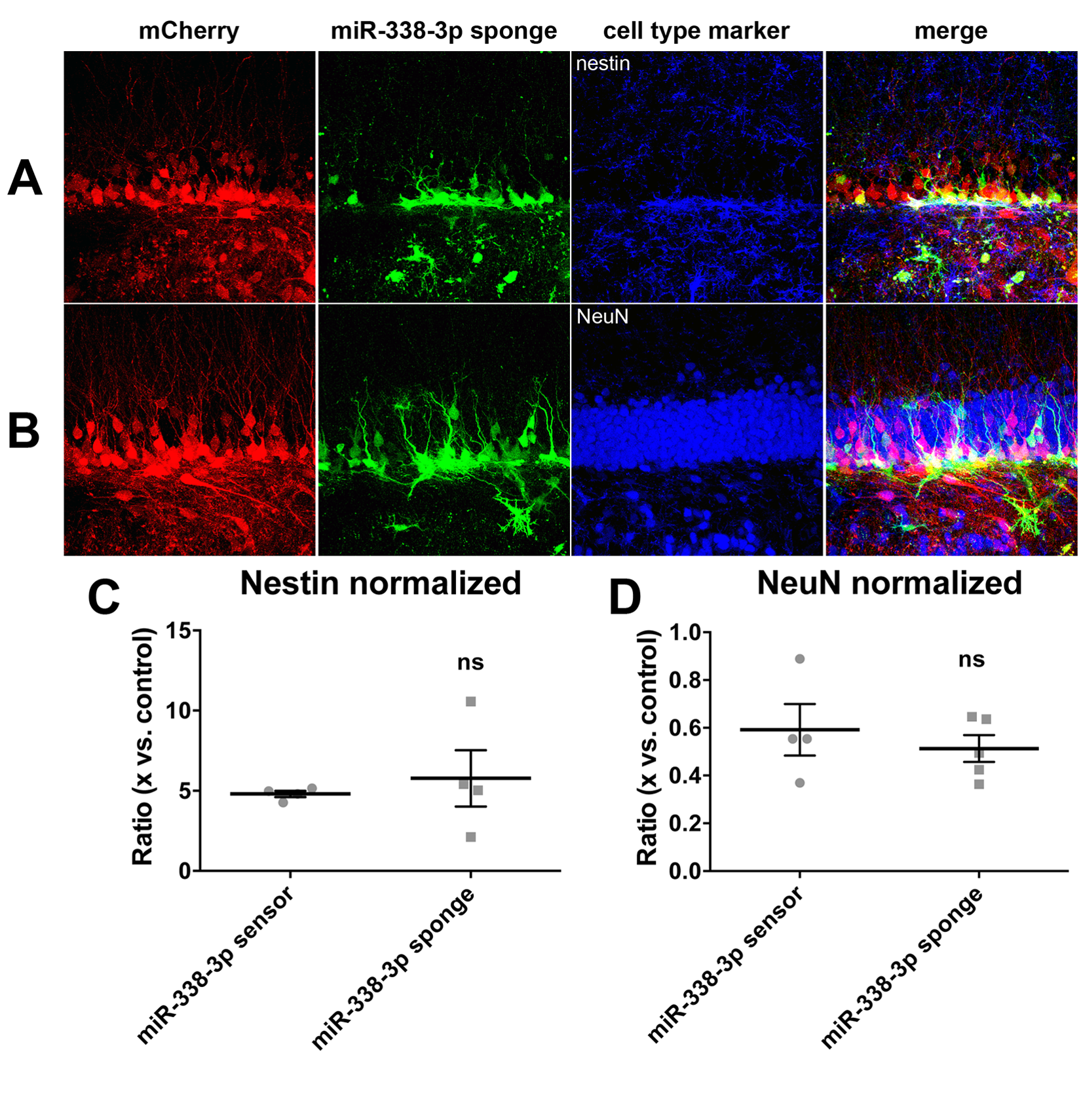

Supplement: S1 Fig — A. Representative images displaying co-localization of nestin (blue) with an mCherry control virus (red) and the miR-338-3p sponge virus (green). B. Representative images displaying co-localization of nestin (blue) with an mCherry control virus (red) and the miR-338-3p sponge virus (green). C. Ratio of co-labeling of cells by nestin and either the miR-338-3p sensor (n = 4) or the miR-338-3p sponge (n = 4) normalized to respective co-expressed mCherry and nestin co-labeling. D. Ratio of co-labeling of cells by NeuN and either the miR-338-3p sensor (n = 4) or the miR-338-3p sponge (n = 5) normalized to respective co-expressed mCherry and NeuN co-labeling. ns p>0.05, one way ANOVA. Results show mean ± SEM. (TIF) [file pone.0177661.s001.tif]

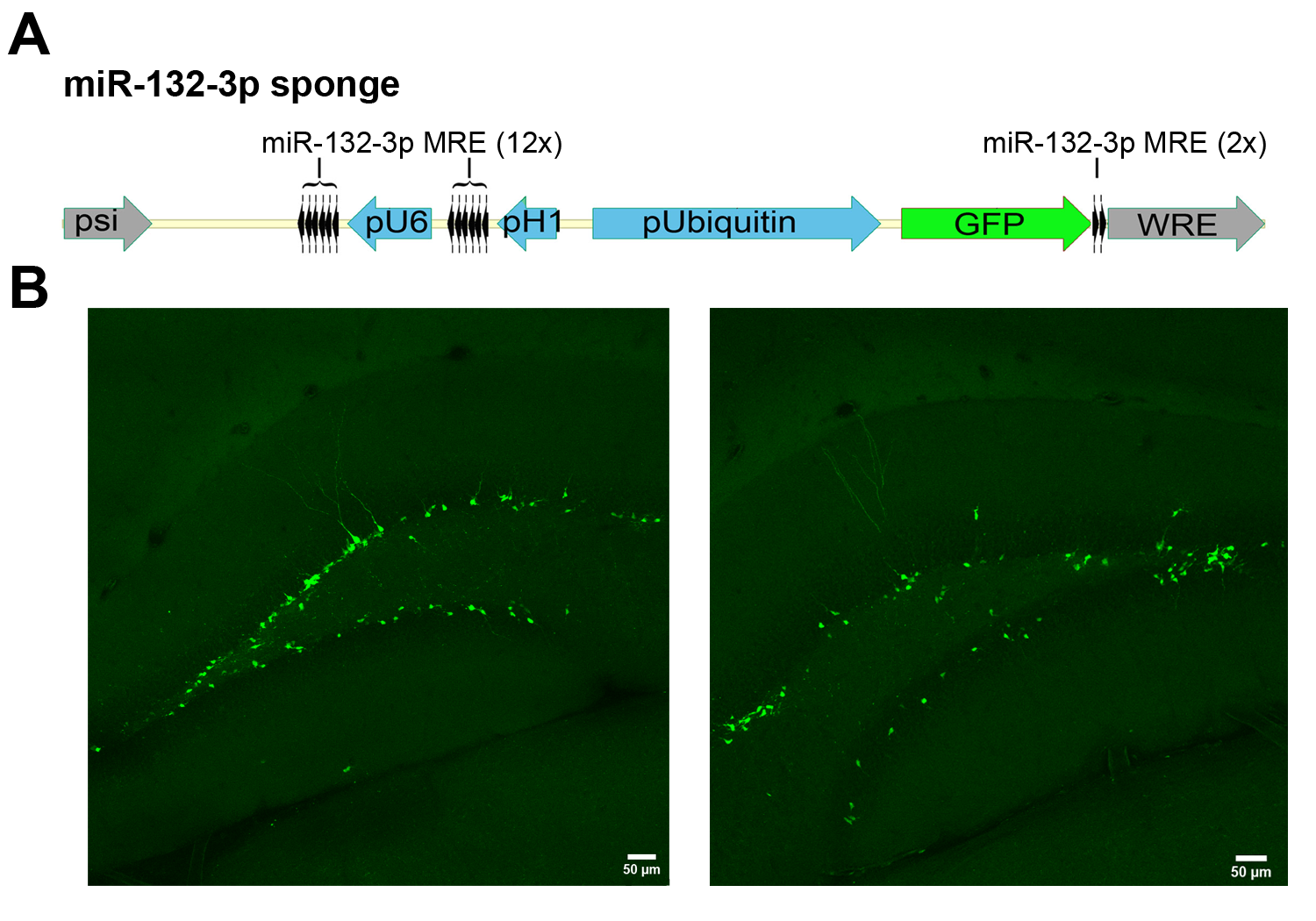

Supplement: S2 Fig — A. Design of lentiviral miR-132-3p sponge with a sensor cassette, using the same vector backbone as the miR-338-3p sponge. The miR-132-3p sensor cassette contains 2 perfectly complementary miR-132-3p target sequences downstream of GFP driven by the pUbiquitin promoter and the sponge cassette consists of 6 targets downstream of both the H1 and U6 promoters for a total of 2 sensor targets to sense miR-132-3p activity and 12 sponge targets to sequester endogenous miR-132-3p. B. Dentate gyrus histology at 7 DPI after miR-132-3p sponge injection. The miR-132-3p sponge knocks down miR-132-3p expression in a subset of dentate gyrus neurons, allowing expression of the GFP sensor construct (green). No neoplastic glioblastoma-like histology was observed. (TIF) [file pone.0177661.s002.tif]
